# Expanded Vermiculite-Filled Polyurethane Foam-Core Bionic Composites: Preparation and Thermal, Compression, and Dynamic Cushion Properties

**DOI:** 10.3390/polym11061028

**Published:** 2019-06-11

**Authors:** Hongyang Wang, Ting-Ting Li, Haitao Ren, Haokai Peng, Shih-Yu Huang, Qi Lin, Jia-Horng Lin, Ching-Wen Lou

**Affiliations:** 1 Innovation Platform of Intelligent and Energy-Saving Textiles, School of Textile Science and Engineering, Tianjin Polytechnic University, Tianjin 300387, China; jobwang1990@163.com (H.W.); renhaitaomail@163.com (H.R.); skyphk@163.com (H.P.); jhlin@fcu.edu.tw (J.-H.L.); 2 Tianjin and Ministry of Education Key Laboratory for Advanced Textile Composite Materials, Tianjin Polytechnic University, Tianjin 300387, China; 3 Fujian Key Laboratory of Novel Functional Fibers and Materials, Minjiang University, Fuzhou 350108, China; 4 Department of Chemical Engineering and Materials, Ocean College, Minjiang University, Fuzhou 350108, China; linqi@mju.edu.cn; 5 Fujian Engineering Research Center of New Chinese Lacquer Material, Minjiang University, Fuzhou 350108, China; 6 Laboratory of Fiber Application and Manufacturing, Department of Fiber and Composite Materials, Feng Chia University, Taichung 40724, Taiwan; 7 Department of Fashion Design, Asia University, Taichung 41354, Taiwan; 8 School of Chinese Medicine, China Medical University, Taichung 40402, Taiwan; 9 College of Textile and Clothing, Qingdao University, Qingdao 266071, China; 10 Department of Bioinformatics and Medical Engineering, Asia University, Taichung 41354, Taiwan; 11 Department of Medical Research, China Medical University Hospital, China Medical University, Taichung 40402, Taiwan

**Keywords:** bionic composites, foam, expanded vermiculite, cushion

## Abstract

In this article, expanded vermiculite (EV)-enhanced polyurethane foam bionic composites inspired by pomelo peel is proposed. The columnar lattice structure mold is employed to constitute the periodic interface structure and gradient foam structure, and the nylon nonwoven fabric is combined as the surface layer. The effects of EV content on the thermal, compression, and dynamic cushion properties of bionic composites are investigated. Results show that residual char increases with EV content, which conduces to decrease the release of heat flow. The proposed bionic composite with columnar lattice structure has optimal compressive modulus, energy absorption and dynamic cushion efficacy when 1 wt% EV is added. However, its performance decreases slowly when EV fillers are continuously added because the cell morphology is changed from round to irregular shape and the interfacial adhesion of filler–matrix is weakened. Owing to their unique bionic structure, composites can absorb 99% of the energy impacted by flat impactor within a smaller deformation and achieve 97% absorption efficiency for a hemispheric impactor in cushion test.

## 1. Introduction

Polyurethane (PU) foam has a porous structure, low production cost, easy processing, and good controllability, all of which make it a superior candidate for cushioning materials. Such composites can absorb energy caused by an impact or vibration by breakage, fracture, bending, and friction against porous cell walls, thereby protecting commodities [1]. The addition of nanoparticles, graphene, carbon nanotube, and silica can improve the mechanical properties of PU foam [2,3]. The syntactic foam material can be used for engineering applications across a range of industries, such as mining, marine, transportation, civil, defense, and aerospace, because of its low density, good thermal efficiency, high strength-to-weight ratio, and impact resistance capacity [4]. However, the cell structure and mechanical property of PU foam are dependent on the hydrophilic and hydrophobic properties of the additives, which can affect the interface strength between fillers and PU matrix [5,6]. Moreover, some scholars found that only the micron size fillers can enter the struts of foam cell to form the PU foam skeleton [7,8]. Therefore, the distinction that can be made for the mechanics of foams involving “small” and “large” particles is related to different geometrical configurations for the particles within the foam [9]. The presence of excessive additives also affects nucleation efficiency, which in turn damages the cell morphology of PU foam [10]. The expanded vermiculite (EV) particles are applied for heat and moisture insulation in actual engineering. Qian et al. investigated the thermal stability of the natural vermiculite-reinforced PU nanocomposites [11]. However, few scholars focused on the PU foams filled with expanded vermiculite (EV) particles of micron size in recent years. Furthermore, some scholars used three-dimensional (3D) warp-knitted spacer fabrics and metallic frame to prepare the sandwich-structured PU composites [12,13,14], the design of which increases the weight of the composites and limits their application range.

Pomelos fall from the tree without the fruit being damaged, indicating that the unique structural composition of the peel can dissipate a large amount of impact energy [15]. Inspired by nature, some scholars proposed gradient foam composites with a hierarchical structure that resembles pomelo peel structure by controlling the overlay layers, foaming temperature, and casting mold [16,17,18]. In a recent study, our team proposed fabric-reinforced composites based on the pomelo peel, which are prepared by two-step foaming without columnar lattice structure and combine with different fabric constructs [19]. Although a few scholars employed the gradient structure, no one combined the outer skin of pomelo, gradient foam, and strong interface to simulate the numerous vertical fiber bundles inside the peel. Such fiber bundles are highly bonded with the cell, forming a tenaciously clinging interface that improves the strength and energy absorption of matrix. Thus, the products are absent in the industrial application.

This study aims to propose a new bionic composite with columnar lattice structure inspired by pomelo peel. The nylon nonwoven fabric is combined as the surface reinforcement and different densities of foam are integrated by using the mold of columnar lattice structure. Improvements in the structural performance of the bionic composites are obtained by combining the EV fillers. Furthermore, the effect of EV contents on the thermal behavior, compression properties, and dynamic cushion efficacy of bionic composites are analyzed systematically. Different shape impactors are also applied to investigate the cushioning mechanisms of the bionic composites.

## 2. Materials and Methods

### 2.1. Materials and Preparation

The manufacturing of bionic composites involves the surface, upper, and lower layers. The surface nylon nonwoven fabrics (Far Eastern New Century Corporation, Taiwan) have an areal density of 300 g/m^2^. The upper layer foam is made of a two-liquid-type high-density flexible PU foam composed of polyether polyol and MDI (diphenylmethane 4,4′-diisocyanate) (APEXLON^®^UR-248, Kuang Lung Shing Corporation, Taiwan). The lower layer foam is made of low-density flexible PU foam (polyether polyol and MDI, CST-1076) from Keshengda Trading, China. Expanded vermiculite was purchased from Hebei ShuoBang Mineral Co., China, and prepared from natural vermiculite heated at 500 °C.

**Figure 1:**
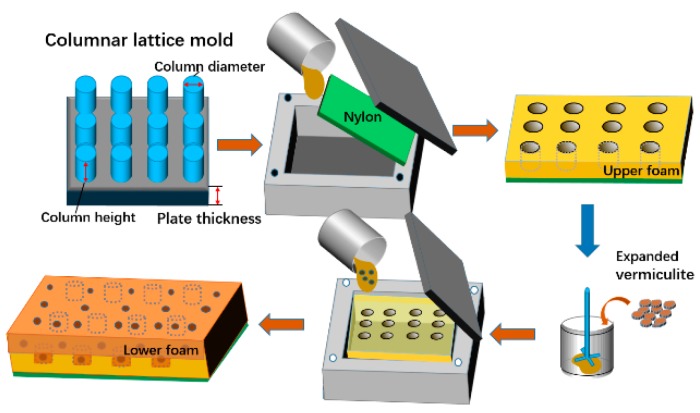
Schematic diagram for the preparation of bionic composites.

The bionic composites were prepared using the two-step foaming technique as follows:

Step 1: For upper layer foam, the polyether polyol and MDI with a weight ratio of 4:1 were blended at 1200 rpm for 15 s at room temperature and normal atmospheric pressure according to the previous literature [20]. Next, the mixture was infused quickly into a square mold (300 × 300 ×10 mm^3^), where a specific mold of columnar lattice structure is applied, and then covered with nylon nonwoven fabric as surface layer before the lid is sealed, as seen in Figure 1. The column diameter was 16 mm, column height was 5 mm, and plate thickness was 5 mm.

Step 2: The product of Step 1 was placed in the square mold instead of the specific mold. Afterwards, the mixture of polyether polyol with various EV contents and MDI were infused before the mold was sealed. The ratio of upper and lower foam volumes was 1:1. In the experiment, different composites were obtained by changing the EV content from 0 wt% to 3 wt%, denoted as NPUH/L, NPUH/L(EV0.5), NPUH/L(EV1), NPUH/L(EV2), and NPUH/L(EV3), where the digit denotes the EV content. Table 1 lists the specifications of the bionic composites.

### 2.2. Testing

**Figure 2:**
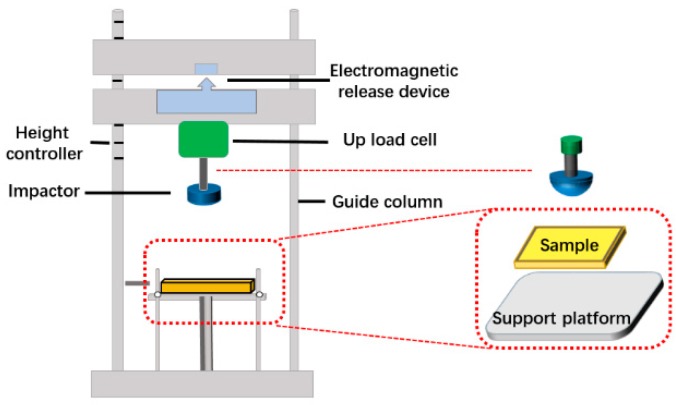
Drop-weight impact tester.

The microstructure of PU foam was observed with a scanning electron microscope (SEM, TM-3030, HITACHI, Tokyo, Japan) at 30 kV. Thermogravimetric analysis (TGA) was performed using TG 209F3 (NETZSCH, Bavaria, Germany) under nitrogen and air atmospheres. Samples with weight of about 5 mg were heated on platinum pans from 50 °C to 700 °C, with the heating rate 20 °C/min. Differential scanning calorimetry was performed using DSC 200F3 (NETZSCH, Bavaria, Germany) under nitrogen and air atmospheres. Samples with weight of about 6 mg were heated from 50 °C to 400 °C with the heating rate 20 °C/min. The Fourier transformed infrared spectroscopy (FTIR Frontier, Nicolet, Thermo Fisher Scientific, Waltham, MA, USA) with an attenuated total reflectance (ATR) accessory under unforced condition was used for the functional group analysis. The FTIR spectra were obtained from 32 scans with a resolution of 4 cm^−1^. A stereomicroscope (SMZ-10A, NIKON, Tokyo, Japan) was used to observe the morphology of the fractured surface of samples in order to analyze the structure. The compression test was implemented at a rate of 10 mm/min using a universal testing machine (HT-2402, HongTa Instrument, Taiwan) as specified in ASTM D1621–16. The drop-weight impact tester applied in cushion test was manufactured by Xin Zhi Electronic Automation Company in Taiwan as shown in Figure 2. The impactor was released from a specified height of 250 mm and drops along a guided column onto the specimen (100 × 100 mm^2^) placed on the anvil. The weight of the impactor was 8 kg and it was made of polished steel with a flat and hemisphere impactor. The contact force applied to bionic composites was studied by measuring the force during the impact test using load cell placed on top of impactor as specified in ASTM D1596-14. Five samples for each specification were used for the tests.

## 3. Results and Discussion

### 3.1. Structural Features of the Pomelo Peel and Bionic Composites

**Figure 3:**
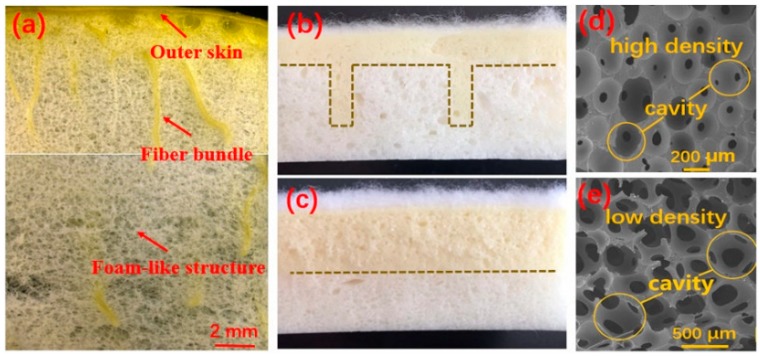
(**a**) Cross section of pomelo peel [21]; (**b**) Cross section of bionic composite with columnar lattice structure; (**c**) Cross section of bionic composite with parallel structure (NPUH-L shows bionic composite with parallel structure); (**d**) Microstructure of high-density polyurethane (PU) foam in upper layer; (**e**) Microstructure of low-density PU foam in lower layer.

As shown in Figure 3a, the cross section of the pomelo peel reveals that that the exocarp is mainly composed of three parts: the foam-like structure, outer skin, and fiber bundles [15]. The large volume fraction of the peel is taken by internal foam-like structure with a gradual increase in the cell size from the outside toward the inside. Furthermore, fiber bundles are distributed periodically from the outer skin towards the foam-like structure. Figure 3b,c show that two kinds of PU foam bionic composites with different interfacial structure are prepared by the two-step foaming, following the structure of pomelo peel. The structure of Figure 3b is named columnar lattice structure and that in Figure 3c is named parallel structure. These bionic composites are composed of the nylon nonwoven fabric as the surface layer, high-density PU foam as the upper layer, and low-density PU foam as the lower layer. The microstructure of PU foam is a gradient structure as shown in Figure 3d,e; the cavity of high-density PU foam is 220 μm and the case for low-density PU foam is 600 μm.

### 3.2. Quasi-Static Compression and Dynamic Cushioning Properties of the Pomelo Peel and Bionic Composites

**Figure 4:**
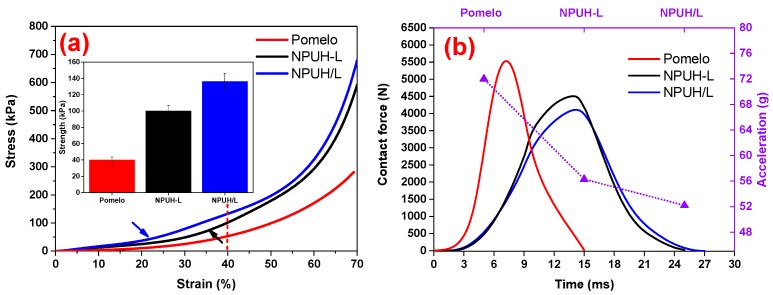
(**a**) Strain–stress curves of the pomelo peel and bionic composites; (**b**) Impact signal of the pomelo peel and bionic composites.

**Figure 5:**
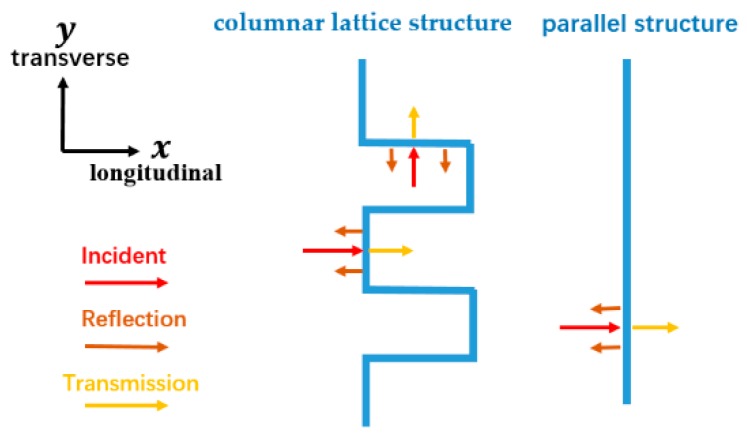
Reflection and transmission of plane stress waves on the interface.

Figure 4a indicates that bionic composites with the columnar lattice structure have the greatest compression resistance compared to the pomelo peel and parallel structure. Moreover, a more abrupt transition appears (indicated by the arrow) in the curves of the bionic composites. The main reason for this phenomenon is the interfacial structure. The columnar lattice structure leads to an earlier transition as demonstrated in Figure 4a. The compression strength of the pomelo is only 40 kPa at the strain of 40%; the case for parallel structure and columnar lattice structure is 100 and 136 kPa, respectively. The impact signal of Figure 4b shows that the conclusion for dynamic cushion is consistent with the quasi-static compression. The contact force of the pomelo peel reached 5553 N within 7.14 ms, bionic composite with parallel structure reached 4498 N within 13.99 ms, and the columnar lattice structure reached 4115 N within 14.26 ms. The results indicate that the bionic composite with columnar lattice structure absorbed more impact energy of the impactor according to the theorem of momentum [22], resulting in the attenuation of the impact force between the impactor and steel plate and prolongation of impact duration. The propagation characteristic of stress wave in the foam medium with columnar lattice structure, except for the wave front, is different from that of the parallel structure. The transmission of wave stress in the interfaces of the columnar lattice and parallel structure are different from the case of a homogeneous foam medium. The reflection and transmission of plane stress waves on the interface are shown in Figure 5. The interfaces of the columnar lattice can prolong the propagation path of wave stress and increase the reflection from the longitudinal and transverse stress wave, resulting in that the stress wave can be more fully absorbed and transformed. Furthermore, the wave front in columnar lattice structure was decayed more when compared to the parallel structure. The interface of parallel structure can only reflect the longitudinal stress wave, while the columnar lattice structure can reflect the longitudinal and transverse stress wave, as demonstrated in Figure 5. Hence, the complex propagation path and reflection of transverse stress on the lattice interfaces between upper and lower foam decay the wave front [23]. Furthermore, the minimum acceleration (only 52 g) of the bionic composite with columnar lattice structure also demonstrated the greatest cushioning property.

### 3.3. Effect of EV Content on Morphology of Lower Layer Foam

**Figure 6:**
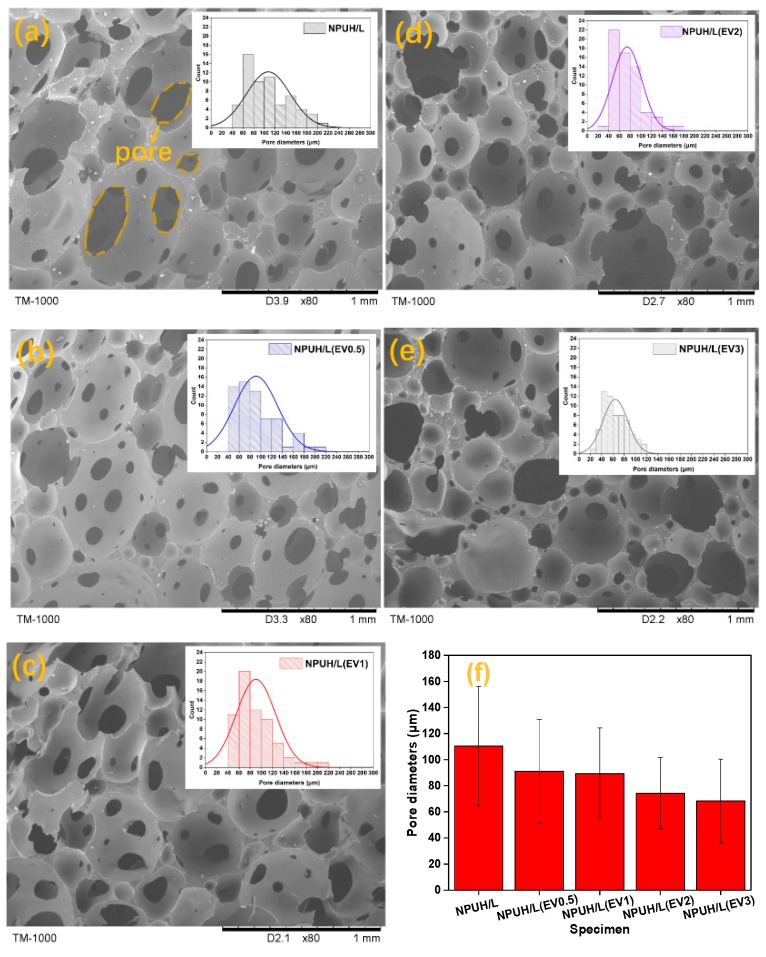
SEM images and pore diameter distribution of lower layer foam with different EV contents. (**a**) NPUH/L; (**b**) NPUH/L(EV0.5); (**c**) NPUH/L(EV1); (**d**) NPUH/L(EV2); (**e**) NPUH/L(EV3); (**f**) the average pore diameters.

When insoluble solid particles are added into the polymer system, a dispersed phase of an insoluble solid occurs, with the exception of the homogeneous gas–liquid system. At this point, both homogeneous and heterogeneous nucleation occur [24]. The bubble nucleus is generated on the interface between the polymer and solid particles when the latter are dispersed in polymer [25]. The microstructure of foams with different EV contents is observed according to the above principles, as shown in Figure 6.

The morphology and pore diameter distribution of lower foams with different EV contents in the same internal hole location are displayed in Figure 6. Figure 6a–e illustrate that the amount and distribution of EV fillers are also crucial factors for obtaining foam cells with a controlled structure and uniform distribution [26]. With the increase in EV content, the pore diameter decreases and the pore distribution narrows, owing to the increased viscosity of polyol. Furthermore, the presence of EV fillers still decreases the energy barrier of cell nucleation and urges the nucleation to occur at the filler–polymer interface [24]. Meanwhile, the increased bubbles start to nucleate concurrently, which causes less gas available for bubble growth [27]. Therefore, the addition of EV particles induces heterogeneous nucleation, provides a large number of nucleation sites, and leads to a small average cell size and narrows cell size distribution. Figure 6f indicates that EV content increases from 0 wt% to 3 wt% and pore sizes decrease from 110 μm to 68 μm. This finding is similar to the results previously reported for PMMA–carbon nanotube nanocomposite foams [28]. However, when the EV content is higher than 1 wt%, excessive EV fillers that served as nucleating agent dramatically increase the heterogeneous nucleation rate, decrease the nucleation time interval, and hence facilitate the almost instantaneous growth of cell size. Furthermore, the nucleation sites are unevenly distributed due to the agglomeration of the excess EV fillers, resulting in the decreased nucleation efficiency and the formation of the nonuniform cell morphology as shown in Figure 6d,e.

### 3.4. Effect of EV Content on Thermal Properties of Composites

**Figure 7:**
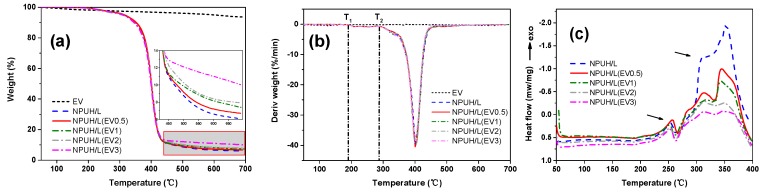
(**a**) Mass loss curves of the reference foam with different EV contents; (**b**) First derivative (DTG) curves of the reference foam with different EV contents; (**c**) Heat release curves of the reference foam with different EV contents.

The effect of EV content on the thermal behavior of the foams was analyzed through TGA and DSC. Figure 7a shows the thermograms of the reference foams with different EV contents and of the neat EV. Figure 7b reveals that the initial degradation temperature (T_1_) takes place at 166 °C. This result corresponds to the decomposition of carbamate group on the main chain of PU at the C–O bond to form isocyanate and polyol. The second degradation temperature (T_2_) takes place at 289 °C, which illustrates that the intermediates (diphenyl ethyl allophanate) continue to decompose into amines, alkene, and CO_2_ [29]. The weight loss around 300 °C corresponds to the decomposition of the hard domain, which takes place at the release of isocyanate, primary and secondary amines, and alcohols. The loss around 400 °C belongs to the degradation of the soft domain, which consists of polyol chains, resulting in the release of a high quantity of carbon dioxide [30]. With the increase in EV content, no significant change occurs in the degradation temperature because EV does not degrade and does not react with PU. The neat EV registers a gradual mass loss between 100 °C and 200 °C, corresponding to the surface and interlayer water, respectively. Table 2 shows the degradation temperatures and the char yield of the foams. A decrease in heat release rate is observed in the second stage of degradation as the EV content increases. This condition is due to the formation of a low thermal conductivity EV char layer on the periphery of foam, which delays the release of heat flow as demonstrated in Figure 7c. This phenomenon is consistent with the results of Gomez-Fernandez et al. [31].

**Figure 8:**
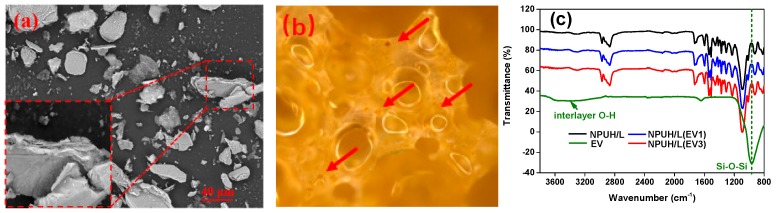
(**a**) Microstructure of EV fillers; (**b**) Photo of bionic composite with EV fillers; (**c**) spectra of bionic composites containing EV fillers.

Figure 8a shows that the EV fillers with lamellar have certain degree of order, and it is not necessary to exfoliate vermiculite into single sheets; this is consistent with the literature [32]. The average particle size calculated by Image Pro Plus software was 21.71 μm. Figure 8b illustrates that EV fillers are wrapped by PU matrix. We can see from Figure 8c that the spectra of bionic composites containing EV did not have any relevant changes, and no new absorptions appeared around 971 cm^−1^ in the spectrum of bionic composites, which corresponds to Si–O–Si groups. Furthermore, this outcome suggests that there were no remarkable chemical reactions between the filler and the polymeric matrix. 

### 3.5. Effect of EV Contents on Compressive Properties of Bionic Composites

**Figure 9:**
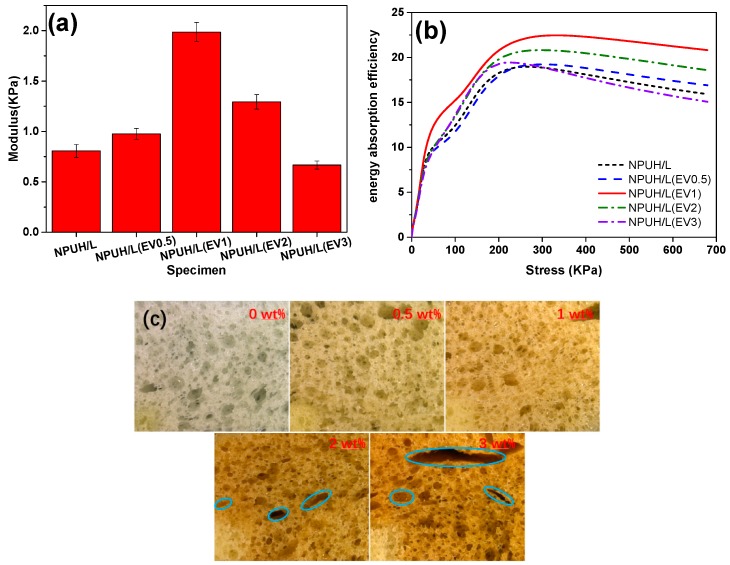
(**a**) Compression modulus of bionic composites with various EV contents; (**b**) energy absorption efficiency curves with various EV contents; (**c**) microstructure of lower foams with various EV contents.

Figure 9a,b show that the changes in compressive modulus and energy absorption efficiency of the bionic composites can be divided into two stages, taking on the trend of rising first and then dropping with the increasing EV content. This finding is consistent with those of previous studies focused on investigating the effects of expandable graphite on PU foams [33]. When a small amount of EV fillers are added, the compressive modulus and energy absorption efficiency of bionic composites increase as a result of the EV fillers being wrapped by PU matrix, which help enhance interfacial adhesion [5]. When the bionic composite is compressed, stress transfers from the matrix to the uniformly dispersed additives. Specifically, 1 wt% of the EV addition showed the best compressive modulus of bionic composites, because of the regular cell morphology and improved interfacial adhesion between EV fillers and PU matrix [34]. In addition, with no more than 1 wt% EV filler, the contact area between fillers and PU matrix increases, which in turn enhances the stress transfer and compression energy absorption efficiency. However, the compressive modulus and energy absorption efficiency decrease slowly when EV fillers are continuously added (>1 wt% EV). This finding is similar to the results previously reported for rigid PU foam filled with glass fiber [33]. This phenomenon can be attributed to two aspects. First, excessive EV fillers are inclined to agglomerate inside the PU matrix, resulting in the decrease of nucleation efficiency and nonuniform cell morphology, as demonstrated in Figure 6. This outcome, in turn, will cause cell morphology to change from a round to an irregular shape, and even to the collapsed cells, as shown in Figure 9c. Second, aggregated EV fillers not only weaken the interfacial adhesiveness but also thin the cell walls, which in turn not totally wrap the additives by the PU matrix [5]. Consequently, the external stress will not be able to transfer through the bionic composites, and the foams around the fillers will be obliged to bear additional stress [6].

### 3.6. Effect of EV Content on the Cushioning Properties of Bionic Composites

**Figure 10:**
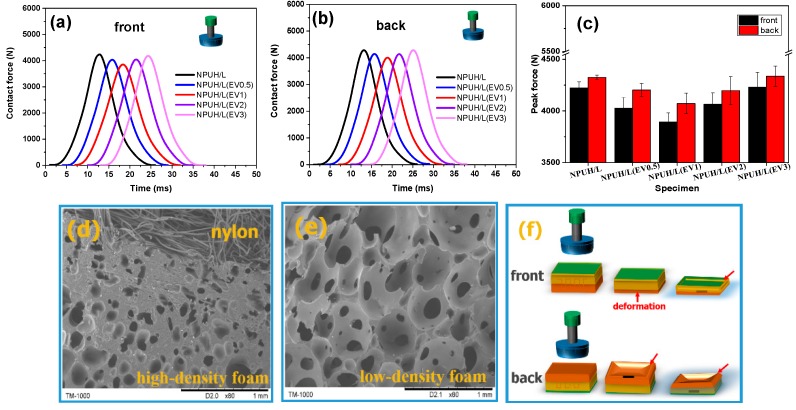
(**a**,**b**) Impact signals of bionic composites with different EV contents; (**c**) Peak contact force of bionic composites with various EV contents; (**d**) The nylon–foam interface of bionic composite; (**e**) The microstructure of low-density foam; (**f**) The diagram of deformation process during impact.

Figure 10a,b show the effect of the EV content on the temporal variations of the contact force. First, the contact force increases slowly when the impactor comes into contact with the bionic composite. When the densification stage reaches, the contact force of the impactor increases rapidly until the impactor is stopped by the steel platform. Then, the contact force decreases. The impact energy is dissipated by compressive deformation of the bionic composites [35]. The kinetic energy of the impactor transforms into the deformation energy and the dissipated energy through the bionic composites [36]. In Figure 10c, a comparison of the peak contact force of the bionic composites with various EV content indicates that the front (the side of nylon nonwoven fabric) and back sides of the bionic composites have the same trend in the impact signal curves. The results show that peak contact force decreases initially and then increases with the EV content increasing. In the dynamic impact process, the lower the dynamic contact force, the longer the impact time, the lower impact acceleration, and the better cushioning capability [35]. In addition, the bionic composite having EV content of 1 wt% has the lowest peak contact force, indicating the bionic composite has better cushioning capability. As shown in Figure 9, compressive modulus and energy absorption efficiency of the bionic composites both decrease when the EV content is higher than 1 wt%. As the EV content continues to increase, the cell morphology becomes more irregular, and interfacial cohesiveness between the EV fillers and PU matrix decreases, which results in attenuation of the energy dissipation capability. Moreover, large amounts of EV fillers begin to aggregate, which attenuates the deformation capacity of foam cell and then affects the stress transfer between the foam cells.

Figure 10c illustrates that the contact force of impact on the front side is lower than that on the back side, caused by the deformation mechanism of bionic composites. Figure 10d,e indicate that the lower layer foam is prone to deformation because of the lower-density foam. Consequently, when the impactor first comes into contact with the nylon fabric layer, the lower layer foam easily undergoes densification. By contrast, when the impact first occurs in the lower layer foam, the nylon fabric layer is depressed without deformation, as shown in Figure 10f. Therefore, the “fabric skin” of the bionic composites plays an important role in energy absorption, a characteristic that is different from the conventional cushion materials. This difference is mainly due to the unique fabric–foam interface having better integrity and greater rigidity, which results in improved compressive strength [37]. In a previous report, the energy absorption capability of pomelo without epidermis attenuates clearly [38]. In the present study, the bionic composites with nylon fabric skin achieve greater cushioning efficiency.

**Figure 11:**
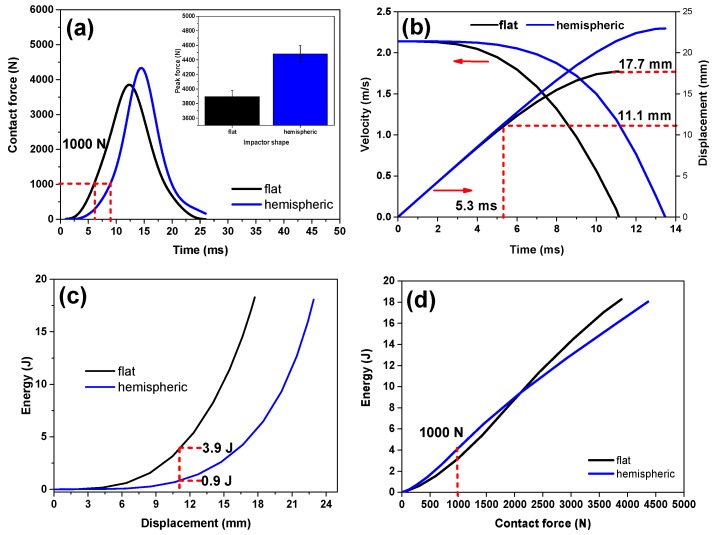
(**a**) Contact force–time curves of bionic composite with 1 wt% EV impacted with different impactors; (**b**) Velocity–displacement curves; (**c**) Displacement–energy absorption curves; (**d**) Contact force–energy absorption curves.

Figure 11 shows that the contact force in the case of hemispheric impactor is bigger compared with that of the flat impactor, reaching 4347 N and 3894 N, respectively, at an EV content of 1 wt% (Figure 11a). The contact area between the flat impactor and bionic composite remains constant, but continuously changes in the case of the hemispheric impactor, resulting in a more complicated impact process [35]. Figure 11b presents a more significant deformation within the longer duration for the hemispheric impactor, indicating that this impactor enables the local foam to reach a stage of greater deformation. By contrast, the impact stops with a shorter time when the flat impactor is used. Mitrevski et al. carried out a similar research and found that the conical impactor has a longer impact duration and bigger depth than does the hemispheric impactor [39]. In the case of the flat impactor, bionic composites begin to enter the densification stage at 5.3 ms, as shown in Figure 11a. Then, the contact force increases rapidly after 5.3 ms. As time passes, the displacement increases slowly and the corresponding impact velocity decreases significantly, as demonstrated in Figure 11b. The bionic composites are more likely to enter the densification stage in the case of the flat impactor. Figure 11c shows that the impact energy from flat impactor is absorbed by bionic composites within a smaller deformation. Bionic composites can absorb 99% of the impact energy of the flat impactor but only 97% of the hemispheric impactor. Figure 11d shows that bionic composites obtain greater energy absorption capability in the case of hemispheric impactor at a contact force of 1000 N. This discrepancy is mainly due to the local structure of bionic composites, which easily reach the densification stage while other parts are still in plateau stage in the case of hemispheric impactor.

## 4. Conclusions

Bionic composites with various EV contents were obtained using the two-step foaming process. The compression, thermal, and cushioning properties were investigated in light of the experimental results. EV contents increased from 0 wt% to 3 wt%, and pore sizes decreased from 110 μm to 68 μm. EV did not react with PU and did not affect the degradation temperature of PU foams under air. However, the release of heat flow decreased as EV contents increased. The changes in compressive modulus, energy absorption efficiency, and cushioning properties of bionic composites can be divided into two stages, taking on the trend of rising first and then dropping with the increasing EV content. The main reason for this outcome is the decrease of nucleation efficiency, resulting in the cell morphology changing from round to irregular shape, and excess fillers are inclined to agglomerate, weakening the cohesiveness of the filler–matrix. In the cushion test, the bionic composite with the columnar lattice structure effectively improved the transmission process of energy dissipation, and the “fabric skin” played an important role in energy absorption. The contact force in the case of the hemispheric impactor was bigger compared with that of the flat impactor. In addition, the bionic composites were more likely to enter the densification stage in the case of the flat impactor. Bionic composites can absorb 99% impact energy of a flat impactor within smaller deformation.

## Figures and Tables

**Table 1:** Specifications of bionic composites.

Sample Code	Surface Layer	Upper Foam	Lower Foam	EV Content (wt%)
NPUH/L	Nylon	high-density	low-density	0
NPUH/L(EV0.5)	Nylon	high-density	low-density	0.5
NPUH/L(EV1)	Nylon	high-density	low-density	1
NPUH/L(EV2)	Nylon	high-density	low-density	2
NPUH/L(EV3)	Nylon	high-density	low-density	3

Note: Expanded vermiculite (EV) is only added to the low-density foam matrix.

**Table 2:** Specifications of composites.

Specimen	EV Content (%)	T_1_ (°C)	T_2_ (°C)	Char Yield (%)
NPUH/L	0	166.7	289.2	6.09
NPUH/L(EV0.5)	0.5	166.5	289.4	6.70
NPUH/L(EV1)	1	166.9	289.3	7.36
NPUH/L(EV2)	2	166.7	289.3	7.91
NPUH/L(EV3)	3	166.7	289.3	10.03
EV	100	-	-	93.62

## References

[1] Loganathan S.B., Shivanand H.K. (2015). Effect of Core Thickness and Core Density on Low Velocity Impact Behavior of Sandwich Panels with PU Foam Core. J. Miner. Mater. Charact. Eng..

[2] Birman V., Chandrashekhara K., Hopkins M.S., Volz J.S. (2013). Effects of nanoparticle impregnation of polyurethane foam core on the performance of sandwich beams. Compos. Part B Eng..

[3] Zhang L., Yilmaz E.D., Schjødt-Thomsen J., Rauhe J.C., Pyrz R. (2011). MWNT reinforced polyurethane foam: Processing, characterization and modelling of mechanical properties. Compos. Sci. Technol..

[4] Pham T.M., Chen W., Kingston J., Hao H. (2018). Impact response and energy absorption of single phase syntactic foam. Compos. Part B Eng..

[5] Sung G., Kim J.H. (2017). Influence of filler surface characteristics on morphological, physical, acoustic properties of polyurethane composite foams filled with inorganic fillers. Compos. Sci. Technol..

[6] Mareri P., Bastide S., Binda N., Crespy A. (1998). Mechanical behaviour of polypropylene composites containing fine mineral filler: Effect of filler surface treatment. Compos. Sci. Technol..

[7] Li T., Chuang Y., Huang C., Lou C., Lin J. (2015). Applying vermiculite and perlite fillers to sound-absorbing/thermal-insulating resilient PU foam composites. Fibers Polym..

[8] Xu W., Wang G., Zheng X. (2015). Research on highly flame-retardant rigid PU foams by combination of nanostructured additives and phosphorus flame retardants. Polym. Degrad. Stab..

[9] Khidas Y., Haffner B., Pitois O. (2015). Critical size effect of particles reinforcing foamed composite materials. Compos. Sci. Technol..

[10] Colton J.S., Suh N. (1987). The Nucleation of Microcellular Thermoplastic Foam With Additives: Part I: Theoretical Considerations. Polym. Eng. Sci..

[11] Qian Y., Lindsay C.I., Macosko C., Stein A. (2011). Synthesis and Properties of Vermiculite-Reinforced Polyurethane Nanocomposites. ACS Appl. Mater. Interfaces.

[12] Zhi C., Long H. (2016). Flexural Properties of Syntactic foam Reinforced by Warp Knitted Spacer Fabric. Autex Res. J..

[13] Caliskan U., Apalak M.K. (2017). Low velocity bending impact behavior of foam core sandwich beams: Experimental. Compos. Part B Eng..

[14] Velosa J.C., Rana S., Fangueiro R., Van Hattum F.W.J., Soutinho F., Marques S. (2012). Mechanical behavior of novel sandwich composite panels based on 3D-knitted spacer fabrics. J. Reinf. Plast. Compos..

[15] Fischer S.F., Thielen M., Loprang R.R., Seidel R., Fleck C., Speck T., Bührig-Polaczek A. (2010). Pummelos as Concept Generators for Biomimetically Inspired Low Weight Structures with Excellent Damping Properties. Adv. Eng. Mater..

[16] Gupta N. (2007). A functionally graded syntactic foam material for high energy absorption under compression. Mater. Lett..

[17] Cao S.C., Liu J., Zhu L., Li L., Dao M., Lu J., Ritchie R.O. (2018). Nature-Inspired Hierarchical Steels. Sci. Rep..

[18] Seidel R., Thielen M., Schmitt C., Bührig-Polaczek A., Fleck C., Speck T. (2013). Fruit walls and nut shells as an inspiration for the design of bio-inspired impact-resistant hierarchically structured materials. Int. J. Des. Nat. Ecodyn..

[19] Wang H., Li T., Wu L., Lou C., Lin J. (2018). Multifunctional, Polyurethane-Based Foam Composites Reinforced by a Fabric Structure: Preparation, Mechanical, Acoustic, and EMI Shielding Properties. Materials.

[20] Yan R., Huang S., Huang C., Hsieh C., Lou C., Lin J. (2016). Effects of needle-punched nonwoven structure on the properties of sandwich flexible composites under static loading and low-velocity impact. J. Compos. Mater..

[21] Li T., Wang H., Huang S., Lou C., Lin J. (2019). Bioinspired foam composites resembling pomelo peel: Structural design and compressive, bursting and cushioning properties. Compos. Part B Eng..

[22] Liu Y., Hu H., Long H., Zhao L. (2011). Impact compressive behavior of warp-knitted spacer fabrics for protective applications. Text. Res. J..

[23] Han C., Sun C.T. (2001). Attenuation of stress wave propagation in periodically layered elastic media. J. Sound Vib..

[24] Zhai W., Yu J., Wu L., Ma W., He J. (2006). Heterogeneous nucleation uniformizing cell size distribution in microcellular nanocomposites foams. Polymer.

[25] Ito Y., Yamashita M., Okamoto M. (2006). Foam Processing and Cellular Structure of Polycarbonate-Based Nanocomposites. Macromol. Mater. Eng..

[26] Chen L., Rende D., Schadler L.S., Ozisik R. (2013). Polymer nanocomposite foams. J. Mater. Chem. A.

[27] Sachse S., Poruri M., Silva F., Michalowski S., Pielichowski K., Njuguna J. (2014). Effect of nanofillers on low energy impact performance of sandwich structures with nanoreinforced polyurethane foam cores. J. Sandw. Struct. Mater..

[28] Zeng C., Hossieny N., Zhang C., Wang B. (2010). Synthesis and processing of PMMA carbon nanotube nanocomposite foams. Polymer.

[29] Norimichi Yoshitake M.F. (1995). Thermal degradation mechanism of α,γ-diphenyl alkyl allophanate as a model polyurethane by pyrolysis- high-resolution gas chromatography/FT-IR. J. Anal. Appl. Pyrolysis.

[30] Pagacz J., Hebda E., Micha Owski S.A., Ozimek J., Sternik D., Pielichowski K. (2016). Polyurethane foams chemically reinforced with POSS—Thermal degradation studies. Thermochim. Acta..

[31] Gomez-Fernandez S., Ugarte L., Pena-Rodriguez C., Zubitur M., Angeles Corcuera M., Eceiza A. (2016). Flexible polyurethane foam nanocomposites with modified layered double hydroxides. Appl. Clay Sci..

[32] Fu Y., Li D., Xu W., Qi Y., Shang W., Wu W., Wang Y. (2014). Applying Vermiculite-Modified Polypropylene Film to Flexible Packaging Material. J. Appl. Polym. Sci..

[33] Cheng J., Shi B., Zhou F., Chen X. (2014). Effects of Inorganic Fillers on the Flame- Retardant and Mechanical Properties of Rigid Polyurethane Foams. J. Appl. Polym. Sci..

[34] Metın D., Tihminlioğlu F., Balköse D., Ülkü S. (2004). The effect of interfacial interactions on the mechanical properties of polypropylene/natural zeolite composites. Compos. Part A Appl. Sci. Manuf..

[35] Liu Y., Au W.M., Hu H. (2013). Protective properties of warp-knitted spacer fabrics under impact in hemispherical form. Part I: Impact behavior analysis of a typical spacer fabric. Text. Res. J..

[36] Wang X.K., Zheng Z.J., Yu J.L., Wang C.F. (2011). Impact Resistance and Energy Absorption of Functionally Graded Cellular Structures. Appl. Mech. Mater..

[37] Wang B., Chen Y., Fan H., Jin F. (2019). Investigation of low-velocity impact behaviors of foamed concrete material. Compos. Part B Eng..

[38] Bührig-Polaczek A., Fleck C., Speck T., Schüler P., Fischer S.F., Caliaro M., Thielen M. (2016). Biomimetic cellular metals-using hierarchical structuring for energy absorption. Bioinspir. Biomim..

[39] Mitrevski T., Marshall I.H., Thomson R., Jones R., Whittingham B. (2005). The effect of impactor shape on the impact response of composite laminates. Compos. Struct..

